# Functional outcome in patients with unstable distal radius fractures, volar locking plate versus external fixation: a meta-analysis

**DOI:** 10.1007/s11751-013-0169-4

**Published:** 2013-07-28

**Authors:** Monique M. J. Walenkamp, Abdelali Bentohami, M. Suzan H. Beerekamp, Rolf W. Peters, Remy van der Heiden, J. Carel Goslings, Niels W. L. Schep

**Affiliations:** 1 Trauma Unit, Department of Surgery, Academic Medical Center Amsterdam, Po Box 22660, 1100 DD Amsterdam, The Netherlands; 2 Faculty of Medicine, Erasmus Medical Center, Rotterdam, The Netherlands

**Keywords:** Meta-analysis, Unstable distal radius fracture, Volar locking plate, Bridging external fixator, Functional outcome

## Abstract

The aim of this study was to compare bridging external fixation with volar locked plating in patients with unstable distal radial fractures regarding functional outcome. A systematic search was performed in the Cochrane Central Register of Controlled Trials, Medline and EMBASE. All randomized controlled trials that compared bridging external fixation directly with volar locked plating in patients with distal radial fractures were considered. Three reviewers extracted data independently from eligible studies using a data collection form. Studies in which the primary endpoint was measured on the disabilities of the arm shoulder and hand (DASH) score at 3, 6 and 12 months were included in the analysis. To this end, mean scores and standard deviations were extracted. The software package Revman 5 provided by the Cochrane Collaboration was used for data analysis. Three studies involving 174 patients were analyzed. Ninety patients were treated with an (augmented) bridging external fixator and 84 with a volar locking plate. Data were analyzed with the random effects model. The robustness of the results was explored using a sensitivity analysis. Patients treated with a volar locking plate showed significantly lower DASH scores at all times. A difference of 16 (*p* = 0.006), six (*p* = 0.008) and eight points (*p* = 0.06) was found at 3, 6 and 12 months follow-up, respectively. Patients treated with a volar locking plate showed significantly better functional outcome throughout the entire follow-up. However, this difference was only clinically relevant during the early postoperative period (3 months).

## Introduction

Fractures of the distal radius are common and account for an estimated 17 % of all fractures diagnosed [1, 2]. Two-thirds of these fractures are displaced and require reduction [3]. Several treatment modalities have been advocated, and decision-making is mainly based on fracture type [4, 5].

One possible surgical treatment method is bridging external fixation. This technique relies on ligamentotaxis to obtain and maintain fracture alignment [6]. However, since the introduction of locking plates, open reduction and internal fixation (ORIF) has become increasingly popular in surgical reduction [7]. This technique provides immediate stable fixation that allows early mobilization [5, 8] and may result in a more rapid recovery and improved regain of function [9]. Conversely, bridging external fixation augmented (with or without additional Kirschner wires) is a less demanding, less invasive and faster procedure. Excellent results have been described for both techniques [10–15]. However, no conclusive evidence has been published favoring ORIF with a volar locking plate over bridging external fixation or vice versa [16].

Margaliot et al. [11] conducted a meta-analysis of studies published between 1980 and 2004 on external and internal fixation of distal radial fractures. They concluded there was not sufficient evidence to support the use of ORIF over external fixation. However, outcome data from a large variety of different techniques of internal fixation were pooled. Studies on both locking and nonlocking implants were included resulting in considerable heterogeneity across studies [11]. More recently, Wei et al. [17] performed a similar meta-analysis comparing functional outcome at 1 year in patients with unstable distal radius fractures. The authors pooled data from 12 randomized and nonrandomized trials on seven different techniques of internal fixation. A secondary subgroup analysis of four studies for volar locking plates revealed a significant difference on the disabilities of the arm shoulder and hand (DASH) score in favor of this technique. Unfortunately, exact DASH scores could not be reported, and therefore, clinical relevance of these differences is difficult to evaluate [18]. Moreover, this analysis included one retrospective study [19] and one trial that compared volar locking plates with closed reduction and percutaneous pinning [20]. The authors emphasized that their results were tempered by a substantial heterogeneity present across studies [17]. However, their significant findings justify further examination regarding the benefits of volar locking plates.

Recent studies on ORIF with volar locking plate have described most benefit in the early postoperative period [21, 22]. In addition to improved functional results at 1 year, a more rapid recovery is of clinical interest as well. Therefore, the primary aim of this meta-analysis was to compare bridging external fixation with volar locked plating in patients with unstable distal radius fractures, regarding functional outcome as measured on the DASH score, at 3, 6 and 12 months follow-up. The secondary aim was to compare grip strength, flexion and extension and radiological parameters at 1 year follow-up.

## Materials and methods

The present study was reported according to the PRISMA guidelines (Preferred Reporting Items for Systematic reviews and Meta-Analyses) [23].

### Eligibility criteria

All randomized clinical trials that compared (augmented) bridging external fixation with volar locking plates in adult patients with unstable distal radial fractures were considered. Publication language was restricted to English and Dutch. Studies that did not clearly define the patient population (unstable distal radius fracture) and thus did not the fine the indication for surgery were not included. Trials that compared different fixation techniques or other implants were not included either. Studies that reported functional outcome on the disability of arm, shoulder and hand score at 3, 6 and 12 months follow-up were included.

### Types of outcome measures

The primary outcome measure of this meta-analysis was a functional outcome defined by the DASH score at 3, 6 and 12 months follow-up. The DASH score is a validated 30-item, self-report questionnaire designed to measure physical function and symptoms in patients with musculoskeletal disorders of the upper limb. Lower scores indicate a better functional outcome. The total scale score ranges from 0 (no disability) to 100 (most severe disability) [24]. The secondary outcome measures of this review were as follows: grip strength measured as a percentage of the uninjured side, flexion and extension in degrees, and radiological parameters including radial inclination, volar tilt, ulnar variance and radial length at a minimal of 1 year follow-up.

### Data sources

We conducted a search for three electronic databases: Cochrane Central Register of Controlled Trials, Medline and EMBASE in March 2013. In order not to miss recently published literature, the use of MESH terms was avoided. The complete search strategy is depicted in Table 1. Additionally, a cross-reference check for the articles of interest was performed.

**Table 1:** Search strategy

*Medline* ((((distal[Title/Abstract]) AND fracture*[Title/Abstract]) AND ((radius[Title/Abstract]) OR radial[Title/Abstract])) OR (((((colles’ fracture*[Title/Abstract]) OR colles fracture*[Title/Abstract]) OR smith fracture*[Title/Abstract]) OR barton fracture*[Title/Abstract]) OR wrist fracture*[Title/Abstract])) AND (((volar[Title/Abstract]) OR palmar[Title/Abstract]) OR palmer[Title/Abstract]) AND ((((external fix*[Title/Abstract]) OR fixation ext*[Title/Abstract]) OR fixateur ext*[Title/Abstract]) OR fixator ext*[Title/Abstract])
*EMBASE* ((((distal.ti,ab) AND fracture*.ti,ab) AND ((radius.ti,ab) OR radial.ti,ab)) OR (((((colles’ fracture*.ti,ab) OR colles fracture*.ti,ab) OR smith fracture*.ti,ab) OR barton fracture*.ti,ab) OR wrist fracture*.ti,ab)) AND (((volar.ti,ab) OR palmar.ti,ab) OR palmer.ti,ab) AND ((((external fix*.ti,ab) OR fixation ext*.ti,ab) OR fixateur ext*.ti,ab) OR fixator ext*.ti,ab)
*Cochrane Central Register of Controlled Trials* (distal:ti,ab,kw and fracture*:ti,ab,kw) AND (radius:ti,ab,kw or radial:ti,ab,kw or “Colles’ fracture*”:ti,ab,kw or “Colles fracture*”:ti,ab,kw or “Barton’s fracture”:ti,ab,kw or smith fracture*:ti,ab,kw or “Smith’s fracture*”:ti,ab,kw or wrist fracture*:ti,ab,kw) AND (“volar”:ti,ab,kw or “palmar”:ti,ab,kw or “Palmer”:ti,ab,kw) AND (extern*:ti,ab,kw or “fixation”:ti,ab,kw or “fixator”:ti,ab,kw or fixat*:ti,ab,kw)

### Study selection

All titles that resulted from the search strategy described above were screened independently by three reviewers. Publications reporting on completely different subjects were identified and excluded. If titles did not provide sufficient information, abstracts were examined. Cohort studies, case studies, comments and current (management) views were excluded. Eligibility with regard to the in- and exclusion criteria of the remaining articles was subsequently assessed based on full text. Disagreement was resolved by means of discussion which included a second trauma surgeon with a master in clinical epidemiology (NS).

### Data extraction

Three reviewers extracted data independently from eligible studies using a data collection form. Items include study type, number of subjects, patient characteristics, fracture types, treatment method, length of follow-up and outcome measures. Means and standard deviations were extracted for continuous outcomes or calculated from confidence intervals. Studies in which these values were not reported were excluded [15]. If multiple treatment types were studied, only data regarding patients treated with bridging external fixation or ORIF were extracted. Risk of bias was assessed using the GRADE guidelines [25].

### Data synthesis

The software package Revman 5 provided by the Cochrane Collaboration was used for data analysis [26]. The mean differences in DASH scores between treatment groups at 3, 6 and 12 months were calculated with 95 percent confidence intervals. The random effects model was used to pool data [27]. Heterogeneity was explored using the chi-square test, with significance set at *p* < 0.1. For quantification, *I*^2^ was used with values less than 30 % indicating low heterogeneity [28, 29].

### Sensitivity analysis

The stability of the results regarding the DASH scores at 3, 6 and 12 months was tested using a sensitivity analysis under different assumptions. Sensitivity analyses were performed based on methodological quality of the included studies and the meta-analytic model. In addition, the robustness of results was explored by consecutively excluding one study.

## Results

### Literature search

**Fig. 1:**
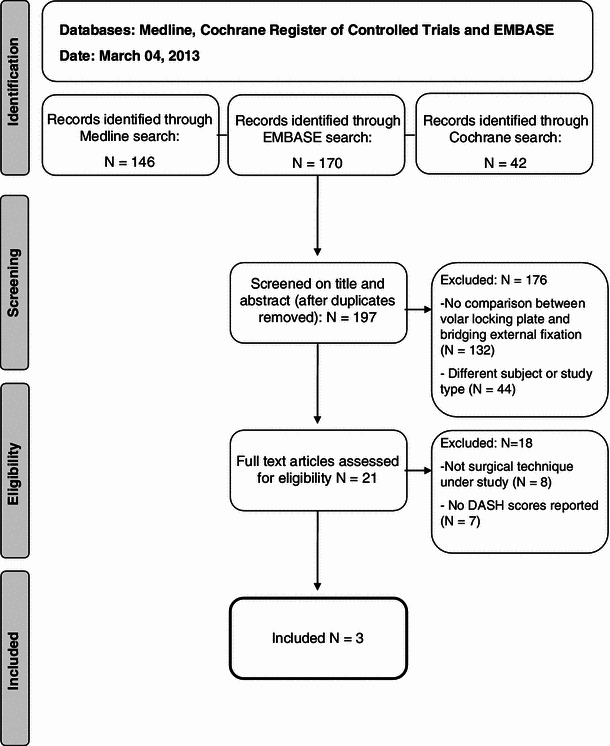
Flow diagram of in- and excluded studies

The search yielded 197 results, three of which met our inclusion criteria (Fig. 1) [30–32]. In total, 174 patients were included, of which 90 were treated with an (augmented) bridging external fixator and 84 patients with a volar locking plate.

### Description of included studies

The study characteristics are summarized in Table 2.

**Table 2:** Details of included studies

Author	Study design	AO classification of included fractures	Sample size		Mean age (years)	Country	Year published	DASH reported at
			Fix ex	Vo. Lo. plate				
Egol et al.	RCT^a^	A, B, C	38	39	51	USA	2008	3, 6, 12 months
Wei et al.	RCT	A3, C1, C2, C3	22	12	57	USA	2009	3, 6, 12 months
Wilcke et al.	RCT	A, C1	30	33	56	Sweden	2011	3, 6, 12 months

^a^Randomized controlled trial

Egol et al. [31] randomized 88 patients with an unstable distal radial fracture to undergo either bridging external fixation (EBI, Parsippany, New Jersey or Stryker, Mahwah, New Jersey) and a K-wire construct or ORIF with a volar locking plate (Hand Innovations, Miami, Florida or Stryker). Inclusion criteria were as follows: loss of reduction following closed reduction and cast immobilization, open fractures or anticipated fracture instability. Criteria for an adequate reduction measured on conventional X-rays included residual dorsal angulation of <10° and loss of radial height of <2 mm. Randomization was performed with a random number generator. The result was handed in a sealed envelope to the treating physician. Seventy-seven patients were included in the analysis, 38 received external fixation with supplementary K-wires and 39 a volar locking plate. DASH scores were reported at a follow-up of 3, 6 and 12 months.

Wei et al. [33] randomized 46 patients with an unstable distal radius fracture to be treated with augmented external fixation (*n* = 22), a volar locking plate (*n* = 12) or a radial locking column plate (*n* = 12). Fractures were considered unstable if fracture fragments were redisplaced following closed reduction and cast immobilization, or if three of the following criteria were met: dorsal angulation of >20°, dorsal comminution, an intra-articular fracture, an associated ulnar styloid fracture or age >60 years. Patients were randomized into three study arms in two phases. First, patients were assigned to be treated with augmented external or internal fixation. During a second randomization, the patients who had been assigned to receive internal fixation were further randomized to be treated with either a volar locking (EBI OptiLock, Parsippany, New Jersey) or a radial locking column plate. Randomization was done by computer-generated allocation using sealed, opaque envelopes. Only data on patients treated with an external fixator or with a volar locking plate were included in this meta-analysis. Treatment with external fixation (Hoffmann II Compact, Stryker) was augmented with K-wires in all patients, additional small buttress plates (*n* = 2) or filling of the metaphyseal void with cancellous bone allograft (*n* = 4) as deemed appropriate by the surgeon. Two patients who had originally been assigned to be treated with a volar locking plate received additional fixation with a dorsal plate, and four patients received supplemental bone grafting following fixation with a volar locking plate. These patients were included in the analysis in the group they were originally assigned to. DASH scores were reported at a follow-up of 3, 6 and 12 months.

Wilcke et al. [32] randomized 63 patients under the age of 70 into volar locking plating (*n* = 33) or bridging external fixation (*n* = 30). Only dorsally displaced AO type A and C1 fractures with an axial shortening of ≥4 mm or a dorsal angulation of ≥20° were included. Randomization was performed by a sealed envelope procedure. Randomization was conducted in blocks of 20 with age stratification set on 50 years. Patients were treated with a volar locking plate (Königsee; Swemac, Sweden) or an external fixator (Hoffmann II Compact, Stryker). In one patient, additional augmentation with a K-wire was performed. DASH scores were reported at a follow-up of 3, 6 and 12 months.

### Methodological quality

The methodological quality of the included randomized controlled trials was moderate according to the guidelines of the GRADE working group [25]. All studies described the process of allocation concealment. Wei et al. randomized their patients into three study arms in two phases resulting in three treatment groups with unequal numbers of subjects. Patients were not blinded since the treatment involved a surgical procedure. Completion of follow-up at 1 year was 78 % in Wei’s study and 100 % in the two other included studies.

In the study by Wei et al., all patients were analyzed based on the intention to treat principle. Egol et al. did not clearly describe crossover to other treatment arms and the type of analysis applied. In the study by Wilcke, one patient in the external fixator group was reoperated and received a supplementary volar plate. This patient was analyzed in the external fixator treatment arm. Power calculations were done for all three trials.

### Functional and radiological outcome

**Fig. 2:**
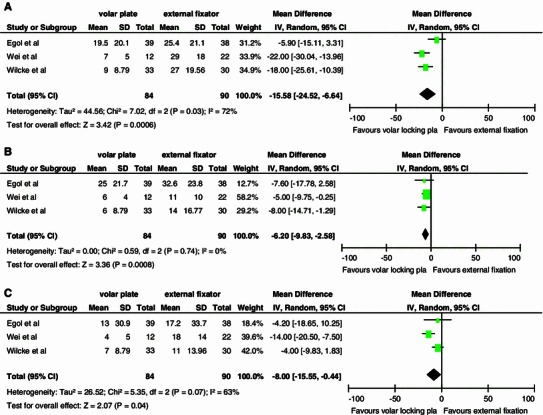
DASH scores at 3, 6 and 12 months. **a** Table and forest plot illustrating functional outcome based on DASH scores comparing external fixation with a volar locking plate at 3 months with a random effects model. **b** Table and forest plot illustrating functional outcome based on DASH scores comparing external fixation with a volar locking plate at 6 months with a random effects model. **c** Table and forest plot illustrating functional outcome based on DASH scores comparing external fixation with a volar locking plate at 12 months with a random effects model. *SD* standard deviation, *CI* confidence interval, *df* degrees of freedom, *IV* inverse variance

At 3 months follow-up, there was a significant difference of 16 points in DASH score favoring the locking plate (95 % CI −24.52, −6.64). At 6 and 12 months, we found a significant difference of 6 (95 % CI −9.83, −2.58) and eight points (95 % CI −15.55, −0.44), respectively (Fig. 2a–c).

**Fig. 3:**
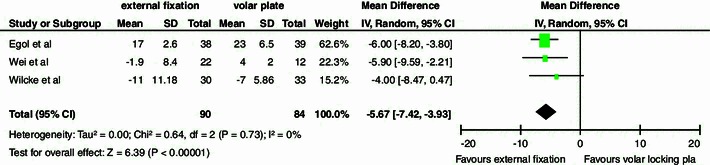
Volar tilt. Table and forest plot illustrating radiographic outcome based on volar tilt comparing external fixation with a volar locking plate at 12 months with a random effects model. The found difference of six degrees indicates a more accurate anatomical reconstruction of the volar tilt after treatment with a volar locking plate. *SD* standard deviation, *CI* confidence interval, *df* degrees of freedom, *IV* inverse variance

A significant difference in volar tilt was observed in favor of treatment with a volar locking plate (Fig. 3). No significant differences were demonstrated in the other secondary outcomes (Table 3).

**Table 3:** For the secondary outcomes such as grip strength, flexion, extension, radial inclination, ulnar variance and radial length, no significant differences were demonstrated

Outcome	Number of studies	Mean difference
Grip strength as percentage of uninjured side	3	−1.73 (−12.27, 15.73)
Flexion (degrees)	2	0.44 (−4.66, 5.53)
Extension (degrees)	2	4.46 (−5.21, 14.14)
Radial inclination (degrees)	2	−2.06 (−4.6, 0.49)
Ulnar variance (mm)	3	−0.086 (1.82, 0.10)
Radial length (mm)	3	−0.96 (−1.96, 0.04)

### Sensitivity analysis

Based on methodological quality, the study by Egol et al. was first excluded since they used a per protocol analysis. Subsequently, the trial by Wei et al. was excluded because of their considerable lost to follow-up. These analyses did not alter the findings or conclusions; all differences remained significant. This was similar when the meta-analytic model was changed. Considerable heterogeneity was found in the analysis of DASH score at 3 and 12 months. Data were homogenous for the DASH score at 6 months (*I*^2^ = 0 %). When the study by Egol et al. was excluded, data were homogenous (*I*^2^ = 0 %) for the analysis of DASH score at 3 months as well. The same was witnessed for the DASH score at 12 months when the trial by Wei et al. was excluded.

### Complications

**Fig. 4:**
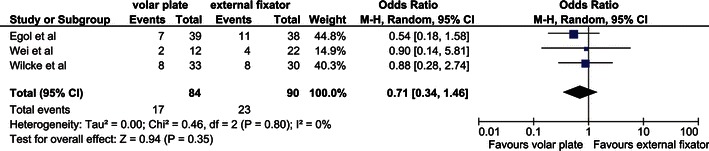
Complications. Table and forest plot illustrating the complication rate comparing treatment with external fixation with a volar locking plate with a random effects model. *CI* confidence interval, *df* degrees of freedom, *M–H* Mantel–Haenszel

A complication rate of 26 % in the external fixator group and 20 % in the volar locking plate group was found (Table 4). These differences were not significant (Fig. 4).

**Table 4:** Complications

Complication	ORIF with volar locking plate (*N*)	Bridging external fixator (*N*)
Pin tract infection		9
Deep infection	1	
Ruptured extensor/flexor pollicis longus tendon	3	1
CRPS I^a^		3
Nonunion	1	1
Painful retained hardware	4	
CTS^b^	2	
Tenolysis for postoperative stiffness		1
Malunion		4
Tendinitis	1	1
Total	17/84 (20 %)	23/90 (26 %)

^a^Complex regional pain syndrome type 1

^b^Carpal tunnel syndrome

## Discussion

This meta-analysis revealed a better functional outcome in patients with unstable distal radius fractures treated with a volar locking plate compared with (augmented) external fixation at 3, 6 and 12 months follow-up. Patients treated with a volar locking plate showed faster rehabilitation reflected in a 16-point difference in DASH score at 3 months. This difference subsided at 6 and 12 months to six and eight points, respectively.

However, in order to fully appreciate these finding, the clinical relevance of the differences in DASH scores should be taken into consideration. The minimal clinically important difference is the smallest difference in an outcome score that a patient perceives as beneficial. In patients with wrist pathology, the minimal clinically important difference in DASH score ranges between 10 points and 15 points [34, 35]. Therefore, functional outcome at 3 months can be considered to be both significantly better and clinically relevant for patients treated with a volar locking plate.

Although considerable heterogeneity was found in the analysis of DASH scores at 3 and 12 months, the differences remained significant under the sensitivity analyses. No clinical or methodological issues could be identified explaining this heterogeneity.

Another significant difference between treatment methods was a slightly improved anatomical restoration of the volar tilt in the ORIF group. The mean difference between external fixation and volar locking plate was six degrees, which indicates a more accurate anatomical reconstruction. Nevertheless, we should keep in mind that radiographic parameters are surrogate endpoints and their clinical relevance remains disputed [36, 37].

There are several strengths to this meta-analysis which include the comprehensive search of the literature and the inclusion of similar trials. Studies in which implants other than volar locking plates, e.g., the fragment-specific wrist fixation system, nonlocking plates or a combination of volar and dorsal plating were used, were not included [14, 20, 38–41]. Similarly, studies using a different form of external fixation and studies with an unclear definition of unstable fractures were excluded as well [20]. Therefore, the results of this meta-analysis will most likely reveal the true magnitude and direction of the differences between the treatments under study.

However, the results of this study should be interpreted with caution because of the following limitations. The power of this meta-analysis was limited since the sample size of the included studies was relatively small. Moreover, the three trials included various AO fracture types and used different definitions of fracture instability and therefore indication for surgery. Finally, unfortunately, only three trials could be included in this analysis. Nevertheless, the quality of a meta-analysis is often considered to be more susceptible to heterogeneity present across studies than the number of included trials [42, 43]. After all, pooled results can be obtained from as few as two studies.

A traditional argument in favor of ORIF with a volar locking plate is early mobilization, which theoretically results in less muscle weakness and therefore improved regain of wrist function. Additionally, the locking principle provides a more rigid construction in the subchondral area of the distal radius, especially in patients with osteoporosis. This theory is in accordance with the results of the current meta-analysis that revealed a significant and clinically relevant improved patient-reported functional outcome for volar locking plate at 3 months. This difference remained significant under a sensitivity analysis and can therefore be considered to be robust. A more rapid recovery might benefit high demanding patients or athletes, and therefore, treatment with volar locking plate for these types of patients with an unstable distal radius fracture is recommended.
